# The Ripple Effect: Quality of Life and Mental Health of Parents of Children with Attention Deficit Hyperactivity Disorder in Saudi Arabia: A Cross-Sectional Study

**DOI:** 10.3390/children11060678

**Published:** 2024-06-03

**Authors:** Shuliweeh Alenezi, Samah H. Alkhawashki, Muneera Alkhorayef, Sarah Alarifi, Shahad Alsahil, Renad Alhaqbani, Nouf Alhussaini

**Affiliations:** 1 Department of Psychiatry, College of Medicine, King Saud University, Riyadh 11451, Saudi Arabia; salenizi@ksu.edu.sa (S.A.);; 2 SABIC Psychological Health Research and Applications Chair (SPHRAC), Department of Psychiatry, College of Medicine, King Saud University, Riyadh 11451, Saudi Arabia

**Keywords:** ADHD, parents, mental health, parenting challenges

## Abstract

Introduction: Attention Deficit Hyperactivity Disorder (ADHD) is a prevalent neurodevelopmental condition in children in Saudi Arabia. ADHD significantly impacts children and their families, particularly by increasing parental stress and diminishing quality of life. In Saudi Arabia, there is a research gap regarding the quality of life and coping mechanisms of parents managing children with ADHD. This study assesses levels of depression and anxiety, quality of life, and coping strategies among parents of children diagnosed with ADHD. Methods: We conducted a cross-sectional online survey with 151 parents of ADHD-diagnosed children, utilizing the WHOQOL-Brief for life quality, the Brief-COPE for coping strategies, and the Patient Health Questionnaire (PHQ) for depression (PHQ9-9 items) and generalized anxiety (GAD7-7 items) modules. Results: Among the parents surveyed, 36% reported moderate to severe depression, while 39.1% experienced moderate to high anxiety levels. Quality of life was significantly positively correlated with higher household monthly income (HHI), employment status, sibling count, and effective coping strategies. Conversely, a parent’s age, educational level, and, in particular, maternal status were inversely related to anxiety levels, with fathers displaying higher maladaptive coping scores. Conclusion: This study sheds light on the considerable anxiety and depression experienced by parents of children with ADHD, significantly affecting their quality of life. Lower quality of life among parents is associated with high levels of depression, anxiety, and ineffective coping strategies. These insights highlight the critical need for interventions to aid parental mental health, thereby improving their overall quality of life amidst ADHD challenges.

## 1. Introduction

The World Health Organization (WHO) identifies Attention Deficit Hyperactivity Disorder (ADHD) as a widely occurring neurodevelopmental condition that affects children’s learning and daily activities. ADHD is characterized primarily by three symptoms: inattention, or a persistent difficulty in sustaining focus; hyperactivity, which involves excessive, situationally inappropriate movements, like constant fidgeting, tapping, or talking; and impulsivity, which entails hasty actions that can be harmful to oneself or others. Individuals with ADHD may exhibit predominantly inattentive or hyperactive/impulsive symptoms, or a combination of both [1].

The Saudi National Mental Health Survey [2] found that among youth and adults aged 15 to 65 years old, the 12-month prevalence of DSM-IV-based criteria for ADHD was 3.2%. It is important to note that there is still to be a national survey that includes younger children up to 15 years. Having said that, there are many individual studies based on different regions in Saudi Arabia, which give region-based prevalences. A recent systematic review and meta-analysis of all observational studies conducted in Saudi Arabia from 2013 to 2021 that included children aged 1 to 17 years old showed a total prevalence of 12.4%. It is important to note that the studies included in the meta-analysis had a high level of heterogeneity and included school-based studies that may not be indicative of the true ADHD prevalence in the whole country [3].

ADHD significantly affects both the individuals diagnosed and their families, creating a complex interplay of stress and behavior. Children’s ADHD symptoms can elevate parental stress, and, in turn, a strained home life may exacerbate ADHD outcomes [4]. Studies have shown that many parents of children with ADHD report increased stress and that they deal with heightened familial and marital discord, parental stress, guilt, susceptibility to depression, increased alcohol use, and diminished quality of life (QoL), especially in emotional and family activity domains [5]. They exhibit less warmth, higher rates of depression and anxiety, and lower WHO Quality of Life (WHOQOL) scores in social relationships and environmental areas, suggesting that ADHD treatment should involve caregivers [6].

Additionally, ADHD is a significant risk factor for non-adaptive coping, emotional, physical, and coordination issues, as well as parental mental health problems. Such parents face more familial issues and are prone to health disorders [7].

A study conducted in Hong Kong noted a significant association of some features, such as severity of hyperactivity/inattention symptoms, presence of a comorbid pervasive developmental disorder, having major medical conditions, lower household income, and a lower educational level, with having lower scores in QoL [8]. Research on parent–child dynamics in relation to ADHD has highlighted the influence of demographic factors, like the child’s age and sex and the parents’ marital and socioeconomic status, on parents’ psychological distress [9]. Along with the challenges of raising a child with ADHD, factors like more siblings, learning difficulties, and comorbid conditions in children with ADHD may exacerbate parental depression and anxiety.

The growing burden of ADHD increases the psychological strain on parents, with mothers of children with ADHD particularly affected by anxiety, depression, and stress [10]. Moreover, parents of children with ADHD have a higher likelihood of depression compared to controls [11]. 

Research combining QoL, depression, anxiety, and coping strategies among these parents is sparse; thus, this study aims to explore how previously reported variables, such as the number of siblings, comorbid learning difficulties, and other psychiatric disorders, affect parents by investigating the impact of having children with ADHD on parents’ QoL, anxiety, depression, and coping strategies. 

## 2. Methodology

### 2.1. Study Design, Participants, and Setting

This is a cross-sectional study that was conducted between August 2022 and November 2022. Participants were parents of children that have been diagnosed by a psychiatrist with any type of ADHD (inattention, hyperactivity, or their combination) based on the diagnostic criteria of DSM-V TR at neurodevelopmental centers in Saudi Arabia. An online survey was created using Google Forms, and participating centers were encouraged to send to their beneficiaries based on their database. 

### 2.2. Measures

- The quality of life scale (World Health Organization Quality of Life—Brief (WHOQOL-Brief) is a 26-item instrument consisting of four domains: physical health (7 items), psychological health (6 items), social relationships (3 items), and environmental health (8 items). It also contains QoL and general health items. Each individual item of the WHOQOL-Brief is scored from 1 to 5 on a response scale, which is stipulated as a 5-point ordinal scale. The physical health domain includes items on mobility, daily activities, functional capacity, energy, pain, and sleep. The psychological domain measures include self-image, negative thoughts, positive attitudes, self-esteem, mentality, learning ability, memory concentration, religion, and mental status. The social relationships domain contains questions on personal relationships, social support, and sex life. The environmental health domain covers issues related to financial resources, safety, health and social services, living physical environment, opportunities to acquire new skills and knowledge, recreation, general environment (noise, air pollution, etc.), and transportation. The Arabic version from the WHO website was used, and it has been demonstrated in different studies to have good reliability and validity [12,13,14].
- The generalized anxiety disorder scale (GAD7) includes seven items calculated by assigning scores of 0, 1, 2, and 3 to the response categories of “not at all”, “several days”, “more than half the days”, and “nearly every day”. The GAD7 total score ranges from 0 to 21, in which “0–4: minimal anxiety”, “5–9: mild anxiety”, “10–14: moderate anxiety”, and “15–21: severe anxiety”.
- The depression scale (PHQ9) is a nine-item self-administered version of the PRIME-MD diagnostic instrument for common mental disorders. The PHQ9 is the depression module, which scores each of the 9 DSM-IV criteria as “0” (not at all) to “3” (nearly every day). Both GAD7 and PHQ9 have been translated and validated for the Saudi population and show great validity and reliability [15].
- The Brief-COPE scale is a 28-item self-report questionnaire designed to measure effective and ineffective ways to cope with a stressful life event. The scale consists of three subscales: Problem-Focused Coping, Emotion-Focused Coping, and Avoidant Coping. Scores are presented for three overarching coping styles as average scores (sum of item scores divided by number of items), indicating the degree to which the respondent has been engaging in that coping style: (1 = I haven’t been doing this at all, 2 = A little bit, 3 = A medium amount, 4 = I’ve been doing this a lot). This tool was shown to be a valid and reliable instrument among the Saudi population [16].

### 2.3. Procedure

Following approval from the International Review Board (IRB) at King Saud University—College of Medicine, the research team contacted all neurodevelopmental disorder clinics across Saudi Arabia, utilizing publicly available information from the Ministry of Health website. Subsequently, an electronic survey was meticulously designed, incorporating socioeconomic variables identified through a comprehensive literature review alongside other instruments: the WHOQOL-Brief for assessing life quality, the Brief-COPE for analyzing coping strategies, and the Patient Health Questionnaire (PHQ) modules for depression (PHQ9-9 items) and generalized anxiety (GAD7-7 items). The survey was disseminated to gather responses from parents of children with confirmed ADHD. Members of the research team oversaw the review and collection of pertinent data from medical charts. Stringent measures were implemented to ensure the security and confidentiality of collected data, with their utilization strictly confined to research objectives.

### 2.4. Internal Consistency and Reliability of the Measurement Questionnaires

The questionnaires were assessed for internal consistency using Cronbach’s alpha test. The analysis revealed that the Brief-COPE questionnaire demonstrated good internal consistency, with a Cronbach’s alpha coefficient of 0.89. Similarly, the PHQ9 questionnaire showed sufficient internal consistency, with a Cronbach’s alpha coefficient of 0.88. The generalized anxiety GAD7 questionnaire also exhibited high internal consistency, with a Cronbach’s alpha coefficient of 0.92. Furthermore, the WHOQOL-Brief, which consists of twenty-six indicators assessing parents’ perceived quality of life, demonstrated excellent internal consistency, with a Cronbach’s alpha coefficient of 0.940 (refer to Table A1 in the Appendix A). These findings suggest that all four questionnaire measures were reliably understood and completed by parents, indicating uniformity in their measurement reliability.

### 2.5. Statistical Analysis

The mean and standard deviation were used to describe the continuous variables, and the median and the interquartile ranges were used to describe variables that showed statistical normality assumption violations. The categorical variables were described with the frequencies and percentages. 

The test of Cronbach’s alpha was used to assess the internal consistency of the measurement questionnaires. Spearman’s (Rho) test of correlation was used to assess the bivariate correlations between metric-measured perceptions. Generalized Linear Multivariable Modeling was used to assess the statistical significance of predictors for each of the measured parental perceptions/concepts (anxiety, depression, and adaptive and maladaptive coping) using the Gamma Regression due to the presence of skewness in the error modeling using the other conventional regression methods. 

The association between the predictor variables and their dependent outcome variables was expressed as multivariate-adjusted Risk Rates (RRs) with a 95% confidence interval. The parental quality of life (QoL) was regressed with the standard multivariable Linear Regression Analysis against parental and child characteristics and ADHD-related factors, and the association between predictor-independent variables with the parental perceived QoL score was expressed as an Unstandardized Beta coefficient with its associated 95% confidence interval. 

The WHOQOL subscale scores and the overall score were transformed into a 0–100 scale according to the author’s scoring manual.

Continuous variables were described using mean and standard deviation, while variables violating the normality assumption were described using median and interquartile range. Categorical variables were summarized using frequencies and percentages. The internal consistency of measured questionnaires was assessed using Cronbach’s alpha. Bivariate correlations between metric-measured perceptions were evaluated using Spearman’s (Rho) test of correlation.

To assess the statistical significance of predictors for each measured parental perception/concept (anxiety, depression, and adaptive and maladaptive coping), Generalized Linear Multivariable Modeling was employed. Due to skewness in error modeling, Gamma Regression was used. The association between predictor variables and dependent outcome variables was expressed as multivariate-adjusted Risk Rates (RR) with a 95% confidence interval.

Parental quality of life (QoL) was regressed using standard multivariable Linear Regression Analysis against parental and child characteristics and ADHD-related factors. The association between predictor-independent variables and the parental perceived QoL score was expressed as an Unstandardized Beta Coefficient with an associated 95% confidence interval.

Statistical data analysis was conducted using IBM SPSS software version 21, while figures and depictions were generated using Microsoft Excel 2019 spreadsheets. A significance level of 0.050 was considered for all tests.

## 3. Results 

### Parental Characteristics and Characteristics of Children with ADHD 

The analyzed findings regarding the sociodemographic characteristics of the measured sample of ADHD-diagnosed children and their parents are displayed in Table 1. 

**Figure 1:**
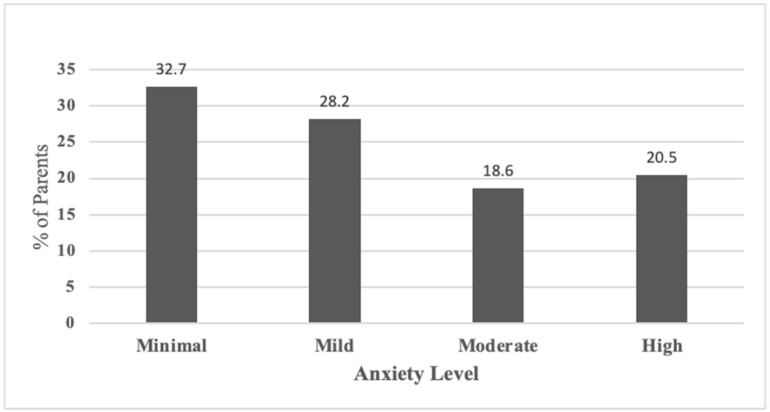
The parents’ perceived anxiety levels.

**Figure 2:**
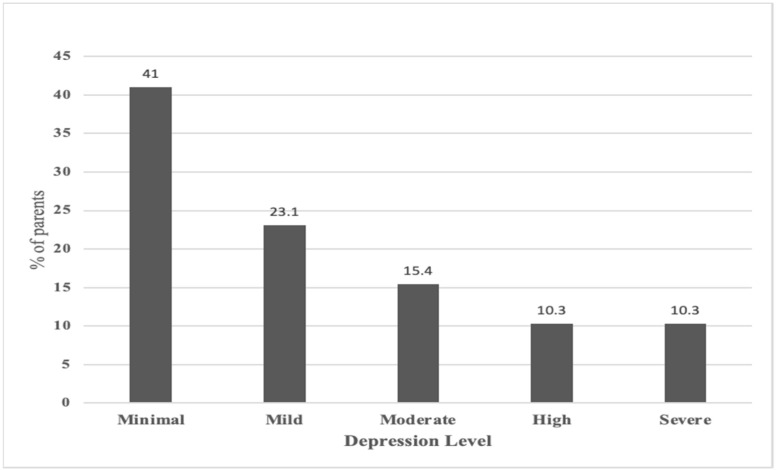
The parents’ perceived depression Levels.

According to the GAD7 scale, 18.6% of parents were considered to have moderate anxiety, and 20.5% had high anxiety levels (Figure 1). Moreover, the PHQ9 scale showed that 23.1% of parents were considered to have mild depression, and 15.4% had a moderate depression level, while 10.3% and 10.3% were found to have high and severe depression levels, respectively (Figure 2). 

The study’s bivariate correlations unveiled several noteworthy insights concerning parents’ perceived quality of life (WHOQOL-Brief) scores and their relationships with various factors, as illustrated in Table 2.

The findings highlighted that parents’ perceived quality of life scores exhibited positive correlations with the subscale scores of physical, psychological, social, and environmental well-being. Additionally, parents’ general life and health satisfaction scores demonstrated positive and significant associations with their mean perceived quality of life scores.

Conversely, significant negative correlations were observed between parents’ mean perceived quality of life scores and their perceived generalized anxiety and depression scores (rho = −0.541, *p* < 0.010, rho = −0.725, *p*-value < 0.010, respectively). These findings suggest that as anxiety levels and depression increased, perceived quality of life scores tended to decrease.

Furthermore, the study revealed that a higher utilization of maladaptive coping strategies among parents of children with ADHD predicted lower perceived quality of life (rho = −0.426, *p*-value < 0.010). Interestingly, no significant correlation was found between parents’ perceived adaptive coping scores and their mean perceived quality of life scores.

Moreover, parents’ mean perceived depression scores exhibited positive correlations with their mean perceived maladaptive and adaptive coping scores (*p*-value < 0.010), indicating that as depression levels increased, both maladaptive and adaptive coping strategies tended to increase.

To gain a deeper understanding of the factors influencing parents’ perceived quality of life, standard multivariable Linear Regression Analysis was employed to regress the parents’ mean perceived overall WHOQOL-Brief score against various sociodemographic characteristics, the outcomes of children with ADHD, and other relevant factors. The resulting findings, presented in Table 3, unveiled several significant correlations.

Firstly, parents’ household monthly income level exhibited a significant and positive correlation with their mean perceived quality of life score. Specifically, parents with a household monthly income of ≥SAR 5000/Month reported significantly higher mean perceived quality of life scores compared to those with a household income of <SAR 5000/Month, with a beta coefficient of 2.482 and a *p*-value < 0.001.

Additionally, parents’ employment status emerged as a significant factor correlated with their mean perceived quality of life score. Employed individuals reported significantly higher mean perceived quality of life scores compared to unemployed individuals, with a beta coefficient of 3.748 and a *p*-value of 0.030.

Unsurprisingly, parents’ mean perceived depression scores (PHQ9) demonstrated a significant negative correlation with their mean perceived quality of life score. Higher levels of depression among parents of ADHD-diagnosed children predicted significantly lower mean perceived quality of life scores, with a beta coefficient of −1.091 and a *p*-value < 0.001.

Furthermore, the parental mean perceived adaptive coping score exhibited a significant and positive correlation with their mean perceived quality of life score. Greater utilization of adaptive coping strategies among parents of ADHD-diagnosed children predicted significantly higher mean perceived quality of life scores, with a beta coefficient of 4.982 and a *p*-value ≤ 0.001.

The number of siblings in the household also demonstrated a significant positive correlation with parents’ mean perceived quality of life score, with a beta coefficient of 0.874 and a *p*-value of 0.035.

In a Multivariable Generalized Linear Gamma Regression analysis examining parental perceived anxiety scores (GAD7), several significant correlations emerged, as detailed in Table 4. Parents’ age and educational attainment displayed significant negative correlations with their mean perceived anxiety (GAD7) scores. With each additional year of parental age, anxiety tended to decrease by a factor of 2.68% on average (*p*-value < 0.001). Moreover, parents holding a diploma degree or higher education reported significantly lower mean perceived anxiety (GAD7) scores, which was 36.4% less than the score of those with a high school or lower education level (*p*-value < 0.001). A noteworthy correlation was observed between parents’ gender. Fathers, on average, reported 19.8% lower mean perceived anxiety (GAD7) scores compared to mothers (*p*-value = 0.030). Interestingly, households with a monthly income below SAR 5000/Month were associated with a 28.5% lower mean perceived anxiety (GAD7) score compared to those earning ≥SAR 5000/Month (*p*-value = 0.003). The severity of ADHD symptoms in children exhibited a significant correlation with parental mean perceived anxiety (GAD7) scores. Parents of children diagnosed with severe ADHD reported significantly higher mean perceived anxiety (GAD7) scores, which was 26.5% higher than the score of those with moderate or less severe ADHD (*p*-value = 0.002). A significant positive correlation was found between parental mean perceived depression scores and their mean perceived anxiety (GAD7) scores.

When applying Gamma Analysis to the parents’ mean perceived depression score (PHQ9), the results presented in Table 5 revealed several significant findings. There was no significant correlation found between parents’ mean perceived maladaptive coping score and their mean perceived depression score (PHQ9). However, a significant positive correlation was observed between parents’ mean perceived adaptive coping score and their mean perceived depression score. On average, for every one-point increase in the mean perceived adaptive coping score, the mean predicted depression score increased by 30.9% (*p*-value < 0.001). A significant negative correlation was found between parents’ mean perceived quality of life score and their mean perceived depression score (PHQ9). For each additional one-point rise in parents’ mean perceived quality of life score, their mean perceived depression score declined by a factor equal to 2.9%, on average (*p*-value < 0.001).

These findings highlight the intricate relationship between coping mechanisms, quality of life, and parental depression levels, shedding light on factors influencing parental mental well-being in the context of raising children with ADHD.

A Gamma Regression employing the generalized multivariate model was utilized to explore parental coping strategies using the mean maladaptive coping score. The findings derived from the analysis, as presented in Table 6, yielded several significant insights. Fathers of children diagnosed with ADHD reported a significantly higher mean maladaptive coping score compared to mothers, measuring 9.7% higher on average (*p*-value = 0.019). Furthermore, parents diagnosed with a history of mental or psychological illness demonstrated significantly higher mean maladaptive coping scores compared to parents without a known history of mental illness. Specifically, their scores were 14.5% higher on average (*p*-value = 0.024). Interestingly, parental perceived anxiety exhibited a significant and positive correlation with their mean perceived maladaptive coping score (*p*-value = 0.004). Higher levels of parental anxiety predicted significantly higher levels of maladaptive coping in general.

The analysis of parents’ mean adaptive coping score (Table 7) revealed several significant findings. The parents’ mean perceived quality of life score (QoL) exhibited a positive and significant correlation with their mean adaptive coping score (*p*-value < 0.001). Higher perceived quality of life among parents predicted significantly higher perceived adaptive coping.

Interestingly, household income did not correlate with parents’ mean perceived adaptive coping. However, their educational level showed a positive correlation, with parents holding a diploma or a higher educational level reporting significantly higher mean perceived adaptive coping scores (13.2% higher) compared to those with a high school or lower educational level, on average (*p*-value = 0.017).

Furthermore, parents’ mean perceived maladaptive coping score positively correlated with their mean perceived adaptive coping score. This suggests that parents may utilize both adaptive and maladaptive coping strategies simultaneously. For each additional one-point increase in parents’ maladaptive coping score, their mean predicted adaptive coping score tended to rise by 30.9%, on average (*p*-value < 0.001).

Additionally, parents’ mean perceived activities of daily living (ADL) difficulties associated with anxiety positively and significantly correlated with their mean perceived adaptive coping score. As parents’ mean perceived ADL difficulties due to anxiety increased by one point, on average, their mean adaptive coping tended to rise incrementally by a factor equal to 11.4%, on average (*p*-value < 0.001). 

The findings also indicated that parents of children with comorbid learning difficulties had significantly lower average maladaptive coping scores compared to parents whose children did not have learning difficulties, with a *p*-value of 0.012. Similarly, parents of children exhibiting autistic traits had significantly lower maladaptive coping scores than parents of children without such traits, with a *p*-value of 0.013.

These findings underscore the complex interplay between various factors, such as parental perceived quality of life, education level, maladaptive coping, and ADL difficulties associated with anxiety, shaping parents’ adaptive coping strategies in the context of raising children with ADHD. 

## 4. Discussion 

Parenting is a continuous, challenging endeavor that places a substantial burden on parents, potentially limiting their ability to maintain the lifestyle they enjoyed before their child’s birth. The behavior of a child with ADHD introduces additional challenges to parenting [9]. This discussion aims to examine the relationship between parental anxiety, depression, and quality of life within the context of ADHD, along with the coping mechanisms parents utilize to manage these challenges.

Our study’s findings indicate that anxiety is a prevalent issue among parents of children with ADHD, with over 39% reporting medium to high levels of anxiety. This aligns with prior research consistently demonstrating that parents of children with ADHD often face increased levels of anxiety [4,10,17]. We observed significant negative correlations between parents’ age and educational attainment and their mean perceived anxiety scores, suggesting that older and more educated parents tend to experience lower levels of anxiety, corroborated by Oguzoncul and colleagues [18]. A notable correlation was also found between parents’ gender, with fathers reporting lower mean perceived anxiety scores compared to mothers. Given that mothers typically serve as the primary caregivers and spend more time with the children, they encounter greater challenges in managing ADHD [19]. While some studies suggest that mothers of children with disabilities experience higher stress levels than fathers [20], other research indicates that fathers are comparably affected [18]. The disparity in mental illness prevalence between mothers and fathers and its impact on children’s functionality requires further investigation.

Notably, there was a strong correlation between the severity of ADHD symptoms in children and the average anxiety scores perceived by their parents. The combined subtype of ADHD was notably linked to higher levels of parenting stress, overall life stress, marital discord, and anxiety about parenting, in contrast to the inattentive subtype [21]. Furthermore, the findings from our study indicated that parents of children with additional learning difficulties or autistic traits generally showed lower maladaptive coping scores than parents of children without such comorbidities. These findings echo qualitative studies highlighting the daily caregiving burdens parents of children with ADHD endure due to their children’s behaviors [22].

Depression is common among caregivers of children with ADHD [23]. Mood disorders were the most prevalent psychiatric disorders among these parents, with major depression rates at 48.1% for mothers and 43.0% for fathers [24]. Our study found that 35% of parents reported moderate to severe depression. Previous studies have identified several risk factors for caregiver depression, including gender, income level, being the sole caregiver, and the absence of a living spouse [23,25,26]. Child-related factors, such as hyperactivity, impulsivity, and the combined type of ADHD, also emerged as significant depression markers [23]. It is crucial for clinical practice to focus on treating depression in caregivers of children with ADHD and to screen for depressive symptoms [25]. The significant impact of depression on parents’ daily functioning and overall well-being underscores the need for targeted interventions and support services.

Quality of life among parents of children with ADHD was a significant focus of our study. Previous research indicates that these parents experience a lower quality of life compared to parents of healthy children [8]. Our use of the WHOQOL-Brief questionnaire to assess parental satisfaction across various life aspects provided valuable insights. Despite reports of moderate overall life satisfaction, parents exhibited high satisfaction with their work capacity, daily activity performance, personal relationships, and support from friends. The strong link between family support and improved outcomes for parents of chronically ill patients is well-documented [27], and our findings reinforce the importance of family support in maintaining the mental health of caregivers of children with ADHD. This contrasts with studies reporting parents’ feelings of isolation and intolerance from others towards their children’s behavior [28]. Furthermore, a study revealed that two-thirds of caregivers of children with ADHD experienced a low quality of life, with all quality of life variables significantly worse compared to those of parents whose children did not have ADHD. Caregivers of children with ADHD who had never worked or had a history of medical conditions reported an excellent degree of self-liking but only a fair level of quality of life and self-competence [7].

Several studies have highlighted that parents of children with ADHD often face more marital conflicts and report lower marital satisfaction than parents of children without ADHD [29]. Interestingly, our study found that parents of children with ADHD reported higher levels of marital satisfaction, underscoring the beneficial impact of social support on their well-being. Yet, it is important to note that these parents expressed lower satisfaction regarding their need for medical treatment to function effectively, pointing to potential physical health challenges stemming from their caregiving roles, which could affect their overall well-being [30].

Our analysis also indicated that household income and employment status significantly influenced perceived quality of life. Parents with higher incomes and those who were employed reported a better quality of life. Furthermore, higher levels of depression and maladaptive coping were associated with a poorer quality of life, whereas adaptive coping correlated with a better quality of life, aligning with previous findings that non-working parents are more adversely affected than their working counterparts [31].

Research on the coping processes among parents of children with ADHD has been limited. This study reveals that fathers of ADHD-diagnosed children scored significantly higher on maladaptive coping strategies (9.7% higher) than mothers. A systematic review by Craig et al. found only two studies addressing the coping strategies of both parents, with one study reporting no significant differences between mothers and fathers in coping strategies [32]. However, the scant representation of fathers in these studies limits our understanding of gender-based differences in coping strategies, highlighting the need for further research with a larger sample of fathers.

Additionally, our findings showed that parents with a prior mental illness diagnosis exhibited significantly higher maladaptive coping scores (14.5% higher) compared to those without a mental health history. These parents may face unique challenges in managing stress related to their parenting role, underscoring the importance of considering parental mental health in coping strategy assessments and support provisions. Stress management for parents of children with ADHD is critical, as stress significantly affects family functioning and, consequently, children’s socioemotional development [33].

Parents engaging in maladaptive coping reported a lower quality of life, whereas those employing adaptive coping mechanisms experienced a higher quality of life. Adopting problem-focused coping strategies, such as seeking information about ADHD, attending workshops or therapy, and establishing structured routines, can empower parents to manage their children’s symptoms more effectively, thus reducing anxiety [34].

Furthermore, this study unveiled intriguing correlations among parental coping, quality of life, anxiety, and depression scores. Parents employing positive coping strategies reported higher depression levels, suggesting that those with adaptive coping strategies might be more conscious of their depressive symptoms. A significant negative correlation was observed between parents’ quality of life scores and their depression scores, indicating that higher quality of life is associated with lower depression levels. Additionally, a positive correlation between parents’ anxiety and depression scores was found, reinforcing the notion that these conditions often coexist and are closely related [35]. This underscores the complex interplay between parental coping mechanisms, mental health, and quality of life in the context of parenting a child with ADHD.

## 5. Conclusions

Living with a child with ADHD presents significant challenges, particularly for parents who must develop specialized coping skills. Our study revealed high levels of anxiety and depression among these parents. Notably, ADHD in children adversely affects their parents’ quality of life, with notable detriments to social well-being and environmental interactions. Our findings indicate that higher levels of depression and anxiety correlate with a lower perceived quality of life among parents. The quality of life score was inversely related to the parents’ maladaptive coping scores; the most frequently reported maladaptive strategies involved self-criticism, self-blame for past events, and verbalizing negative feelings as a release. Furthermore, a greater reliance on maladaptive coping strategies was linked to a diminished quality of life.

In summary, this study underscores the profound effects of depression, anxiety, and coping strategies on the quality of life of parents of children with ADHD. It accentuates the critical need for targeted support and interventions to enhance mental well-being and coping mechanisms within this demographic.

## 6. Limitations

The study delineates specific limitations that could affect the interpretation and generalizability of its findings. Firstly, the small sample size poses a significant constraint, making it difficult to extend the results to a broader population. Secondly, the lack of a control group is a notable limitation. Including parents of children without ADHD could provide a comparative baseline to discern if the observed variables are uniquely prevalent or intensified in the ADHD context. Furthermore, not including the specific characteristics of the children and their symptoms as they pertain to the parents and the effects they may have on the parents’ QoL seems to pose a limitation, and this is a consideration for future exploration. 

Additionally, the methodological design restricts the breadth of possible interpretations due to its inability to assess causality. Without the ability to ascertain the directionality of the effects, it remains unclear whether certain variables, such as depression and anxiety, are outcomes of coping with a child’s ADHD or if they precede and potentially contribute to the perceived challenges of parenting.

Lastly, the cross-sectional nature of the research does not account for the longitudinal impact of environmental factors on the variables studied. This restricts the study’s capacity to understand the evolving nature of parental experiences and the potential for changes in mental well-being, coping strategies, and quality of life over time. Future research could address these limitations by incorporating larger, more diverse samples, control groups, methodological designs capable of assessing causality, and longitudinal approaches to explore the enduring effects of environmental influences. 

## 7. Recommendation

In Saudi Arabia, limited studies have addressed the importance of early intervention for psychological distress in parents of children with ADHD. Conducting more research on prevention methods and psychological screening and assessing the availability and accessibility of mental health services for parents is recommended. Additionally, future studies are needed to investigate the impact of cultural beliefs and religious practices on parental well-being.

## Figures and Tables

**Table 1:** Descriptive analysis of parents’ and children’s sociodemographic characteristics and children’s ADHD-disease-related factors.

	Frequency	Percentage
Relation to the child		
Mother	93	59.6
Father	63	40.4
Age (years), mean (SD)		40.90 (7.32)
Age group		
25–35 years	38	24.4
36–45 years	80	51.3
≥46 years	38	24.4
Educational level		
High school or less	27	17.3
Diploma degree	15	9.6
University degree	82	52.6
Higher studies	32	20.5
Employment status		
Not employed	58	37.2
Employed	98	62.8
Household monthly income (HHI)		
<SAR 5000/M	35	22.4
SAR 5000–10,000/M	43	27.6
>SAR 10,000–15,000/M	42	26.9
More than SAR 15,000/M	36	23.1
Parental separation		
No	138	88.5
Yes	18	11.5
Comorbidity		
No	122	78.2
Yes	34	21.8
What chronic illness?		
Diabetes	10	28.6
Hypertension	8	22.9
Thyroid disease	8	22.6
Skin disease	3	8.6
Other disease	12	34.3
Autoimmune disease	7	20
Have you been previously diagnosed with a mental illness?		
No	140	89.7
Yes	16	10.3
Are you still diagnosed with mental illness?		
No	138	88.5
Yes	18	11.5
Sex of child affected by ADHD		
Female	29	18.6
Male	127	81.4
Age of child affected by ADHD, age (years), mean (SD)		10.03 (3.63)
Number of live siblings, median (IQR)		2 (2)
Does the child receive ADHD treatments?		
No	68	43.6
Yes	88	56.4
Does the child have from any of these disorders? n = 110		
Separation anxiety	4	3.6
Learning difficulties	89	80.9
Autism	31	28.2
Asperger’s syndrome	4	3.6
Residence		
Riyadh city	123	78.8
Other cities	33	21.2

**Table 2:** Spearman’s (Rho) correlations between the parents’ measured perceptions.

	WHOQOL-Brief	Phys	Psych	SOC	ENV	GL	GH	GAD	GAD_DIFF	PHQ	PHQ_DIFF	MADAPT
QOl_brief100 Quality of life (0–100%) score	1.000											
Physical_Dom100 Physical well-being	0.845 **											
Psych_Dom100 Psychological well-being	0.859 **	0.672 **										
Social_Dom100 Social well-being	0.824 **	0.590 **	0.543 **									
Environm_Dom100 Environmental satisfaction	0.833 **	0.612 **	0.713 **	0.611 **								
Qol_1 How would you rate your life?	0.539 **	0.456 **	0.439 **	0.469 **	0.454 **							
Qol_2 How satisfied are you with your health?	0.502 **	0.496 **	0.445 **	0.453 **	0.270 **	0.466 **						
GAD7_score Generalized anxiety disorder score	−0.541 **	−0.566 **	−0.501 **	−0.381 **	−0.353 **	−0.490 **	−0.431 **					
GAD7_DIFF Perceived ADL difficulties associated with anxiety feeling	−0.491 **	−0.568 **	−0.402 **	−0.336 **	−0.356 **	−0.450 **	−0.408 **	0.540 **				
PHQ9_score General health anxiety PHQ9 scale score	−0.725 **	−0.727 **	−0.630 **	−0.575 **	−0.464 **	−0.589 **	−0.617 **	0.789 **	0.640 **			
PHQ9_DIFF Perceived ADL difficulties associated with depression	−0.522 **	−0.558 **	−0.498 **	−0.354 **	−0.378 **	−0.476 **	−0.444 **	0.574 **	0.780 **	0.668 **		
Maladaptive (negative) coping	−0.426 **	−0.445 **	−0.376 **	−0.255 **	−0.304 **	−0.282 **	−0.269 **	0.417 **	0.336 **	0.509 **	0.323 **	
Adaptive (positive) coping	−0.019	−0.061	0.044	−0.058	0.060	−0.126	−0.083	0.151	0.275 **	0.232 **	0.222 **	0.475 **

** correlation is significant at 0.010 level.

**Table 3:** Multivariate Linear Regression Analysis of the parental perceived quality of life (QoL) of parents of children with ADHD (N = 155).

	Unstandardized Beta Coefficients	95% CI for Beta Coefficient		*p*-Value
		Lower Bound	Upper Bound	
(Constant)	66.567	53.262	79.871	<0.001
Age of the parent	−0.144	−0.384	0.097	0.239
Relationship to the child, father	0.485	−3.117	4.086	0.791
Educational level	−1.080	−2.842	0.681	0.227
Household monthly income level ≥SAR 5000/M	2.482	0.889	4.075	0.002
Employment state, employed	3.748	0.375	7.122	0.030
General health anxiety PHQ9 scale score	−1.091	−1.353	0.829	<0.001
Maladaptive coping	−4.369	−7.885	−0.853	0.015
Adaptive coping	4.982	2.468	7.497	<0.001
Number of siblings	0.876	0.064	1.688	0.035
Parent’s history of psychological illness	−3.574	−8.359	1.210	0.142
Child’s ADHD severity level	0.840	−1.667	3.347	0.509

Dependent variable: parental mean perceived overall QoL score. Model’s overall statistical significance: f(11,144) = 18.41, *p*-value < 0.001, model R = 0.764, model adjusted R-squared = 0.553.

**Table 4:** Multivariable Generalized Linear Gamma Regression analysis for parental perceived anxiety (GAD7) score (N = 156) *.

Parameter	Multivariate Adjusted Risk Rate (RR)	95% CI for RR		
		Lower	Upper	*p*-Value
(Intercept)	10.674	4.971	22.917	<0.001
Age of the parent (years)	0.973	0.961	0.986	<0.001
Relation, father	0.802	0.656	0.979	0.030
Household monthly income <SAR 5000	0.715	0.572	0.894	0.003
Educational level, diploma/university/high studies	0.636	0.498	0.812	<0.001
Employment status, employed	1.139	0.935	1.389	0.197
Parental current mental illness, positive	1.219	0.933	1.592	0.147
Child’s ADHD severity level, moderate/high	1.265	1.090	1.467	0.002
Parents’ mean perceived depression (PHQ9) score	1.066	1.050	1.083	<0.001
Parents’ mean perceived (negative) coping score	1.100	0.894	1.352	0.369
Parents’ mean perceived (positive) coping score	1.058	0.913	1.226	0.454

* Dependent variable: parents’ mean perceived anxiety (GAD7) score.

**Table 5:** Multivariable Generalized Linear Gamma Regression analysis for parental perceived depression (PHQ9) score. N = 156.

Parameter	Multivariate Adjusted Risk Rate (RR)	95% CI for RR		
		Lower	Upper	*p*-Value
(Intercept)	10.885	4.089	28.976	<0.001
Age of the parent (years)	0.997	0.984	1.010	0.606
Relation to the child, father	0.903	0.746	1.092	0.294
Educational level	1.127	0.892	1.424	0.316
Employed parent	1.156	0.961	1.392	0.124
Child’s ADHD severity Level, moderate/high	1.091	0.953	1.250	0.206
Parents’ mean perceived (maladaptive/negative) coping score	1.033	0.856	1.246	0.737
Parents’ mean perceived (adaptive/positive) coping score	1.309	1.140	1.503	<0.001
Parental mean perceived quality of life (QoL) score	0.971	0.963	0.979	<0.001
Parental perceived generalized anxiety GAD7 score	1.075	1.055	1.095	<0.001
Household monthly income	0.960	0.883	1.043	0.337

Dependent variable: Patient Health Questionnaire (depression) score.

**Table 6:** Multivariable Generalized Linear Gamma Regression analysis for parental perceived maladaptive coping score. N = 156.

Parameter	Multivariate Adjusted Risk Rate (RR)	95% CI for RR		
		Lower	Upper	*p*-Value
(Intercept)	1.565	1.123	2.180	0.008
Age of the parent (years)	0.996	0.991	1.002	0.219
Relation to the patient, father	1.097	1.016	1.186	0.019
Prior diagnosis with mental Illness	1.145	1.018	1.289	0.024
Sex of the ADHD-diagnosed child, male	0.946	0.883	1.014	0.115
Age of the ADHD-affected child (years)	1.010	0.999	1.020	0.083
Parental mean perceived quality of life (QoL) score	0.993	0.990	0.996	<0.001
Parents’ mean perceived (positive) coping score	1.258	1.193	1.326	<0.001
Parental perceived generalized anxiety GAD7 score	1.010	1.003	1.017	0.004
Having a child with ADHD and learning difficulties	0.912	0.848	0.979	0.012
Having a child with ADHD and autistic traits	0.891	0.813	0.976	0.013

Dependent variable: maladaptive coping score.

**Table 7:** Multivariable Generalized Linear Gamma Regression analysis for parental perceived adaptive coping score. N = 156.

Parameter	Multivariate Adjusted Risk Rate (RR)	95% CI for RR		
		Lower	Upper	*p*-Value
(Intercept)	0.669	0.430	1.040	0.074
Age of the parent (years)	0.999	0.994	1.005	0.763
Relation to the patient, father	0.976	0.898	1.060	0.564
Child receives ADHD treatments/therapy	1.030	0.959	1.107	0.410
Parental mean perceived quality of life (QoL) score	1.009	1.005	1.012	<0.001
Household monthly income <SAR 5000/Month	1.075	0.978	1.183	0.135
Parental educational level, diploma degree or higher	1.132	1.023	1.254	0.017
Parents’ mean perceived (negative) coping score	1.309	1.216	1.410	<0.001
Parental perceived ADL difficulty associated with anxiety	1.114	1.052	1.180	<0.001

Dependent variable: parents’ mean adaptive coping score.

## Data Availability

The data presented in this study are available upon reasonable request from the corresponding author. The data are not publicly available due to privacy reasons.

## Abbreviations

ADHD	Attention Deficit Hyperactivity Disorder
QoL	Quality of life
ADL	Activities of daily living
KSUMC	King Saud University Medical City
WHO	World Health Organization
HHI	Household monthly income

## Appendix A

**Table A1:** The internal consistency analysis of the measured questionnaires.

	Number of Items	Cronbach’s Alpha
Brief-COPE questionnaire	28	0.89
Patient Health Questionnaire (PHQ9)	9	0.88
Generalized anxiety disorder (GAD7) scale	7	0.923
World Health Organization Quality of Life (WHOQOL-Brief) questionnaire	26	0.94
